# High-fat diet from perilla oil induces insulin resistance despite lower serum lipids and increases hepatic fatty acid oxidation in rats

**DOI:** 10.1186/1476-511X-13-15

**Published:** 2014-01-15

**Authors:** Tao Zhang, Shuang Zhao, Wei Li, Lanzhi Ma, Ming Ding, Ruisheng Li, Yuan Liu

**Affiliations:** 1 Laboratory Animal Center of the Academy of Military Medical Science, Beijing 100071, China; 2 Animal Laboratory Center, 302 Hospital of PLA, Beijing 100039, China

**Keywords:** Perilla oil, Alpha-linolenic acid, Serum lipid, Fatty acid oxidation, Insulin sensitivity

## Abstract

**Background:**

The purpose of this study is to investigate the effects of a high-fat diet from perilla oil on serum lipids, hepatic lipid metabolism and insulin sensitivity.

**Methods:**

Male Sprague–Dawley (SD) rats were fed either a control (CT) diet or a diet high in perilla oil (HP). After 16 weeks of feeding, the serum lipids were measured, and the gene expressions involved in hepatic fatty acid oxidation and synthesis were determined. In addition, hepatic fat deposition was detected, and insulin sensitivity was evaluated by means of euglycemic-hyperinsulinemic clamp.

**Results:**

Compared with the rats in the CT group, the HP-feeding significantly decreased the levels of triglyceride (TG), total cholesterol (TCH) and HDL-cholesterol (HDL-c). HP-feeding did not change the levels of LDL-cholesterol (LDL-c), free fatty acid (FFA), intrahepatic lipids or body weight. Moreover, the HP-feeding dramatically increased the mRNA expressions of fatty acid oxidation markers (PPAR-alpha, CPT1A) and fatty acid synthesis markers (SREBP-1, FASN and ACC) in the liver. The HP-feeding induced increased protein levels of CPT1A, while reducing the protein levels of FASN and ACC in the liver. However, the glucose infusion rate significantly increased in the HP group compared with the CT group.

**Conclusions:**

Our data show that, in rats, excessive perilla oil intake may significantly lower serum lipids, strengthen hepatic fatty acid oxidation, and inhibit hepatic fatty acid synthesis, but at the same time may also lead to insulin resistance.

## Introduction

Epidemiological evidence suggests that a western diet rich in fat is associated with obesity, NALFD and insulin resistance [1,2]. In order to investigate the effects of high fat diet on bodies, a large number of experiments have employed high-fat diet-induced animal models [3-5]. A multitude of different high-fat diets have been used with relative fat fractions between 20 and 60% energy as fat, and the basic fat component varies between animal derived fats and plant oils [6]. However, the characteristics of the animal models induced by high-fat diets from various fat sources are different. In our previous experiments, semi-purified diets with fat consisting of 45.73% energy based on animal fats and n-6 fatty acid-containing plant oils led to obesity and NALFD, whereas diets with the same amounts of linseed oil, which were rich in ALA (>55% of total FA), did not [7].

Perilla is one of the richest sources of ALA. ALA and other fatty acids may be transformed into long-chain polyunsaturated fatty acids, such as eicosapentaenoic acid (EPA) and docosahexaenoic acid (DHA), via a series of elongation and desaturation reactions [8]. Delta-6-desaturase catalyzes rate-limiting enzymatic reactions in humans. Two long-chain n-3 PUFAs (EPA and DHA) from fish oil have been widely recognized due to their beneficial effects on health, and are considered as essential supplements in human food [9]. Many studies have shown that n-3 PUFAs play roles in resisting inflammation, increasing insulin sensitivity, and reducing the incidences of obesity and cardiovascular and cerebrovascular diseases [10-12]. In rodents in vivo, n-3 long-chain-PUFA has been shown to have a protective effect against insulin resistance [13]. In general, dietary studies evaluating n-3PUFA efficacy in different model systems rely on low doses [14], in which ~5% fish or flaxseed oil was used as intervention (corresponding to approximately 2–6% of the total energy as n-3PUFAs).

A few studies have tested the effects of n-3PUFAs at higher doses on functional endpoints. Very little is known about the effects of high doses of perilla, which may have a unique therapeutic function, but may also exert negative side effects, thus raising potential safety issues for the general public. The purpose of the current study is to examine the serum lipids, hepatic fat deposition, insulin resistance, hepatic gene expression involving fatty acid oxidation and synthesis in HP-induced rats.

## Results

### Body weight and food consumption

**Figure 1:**
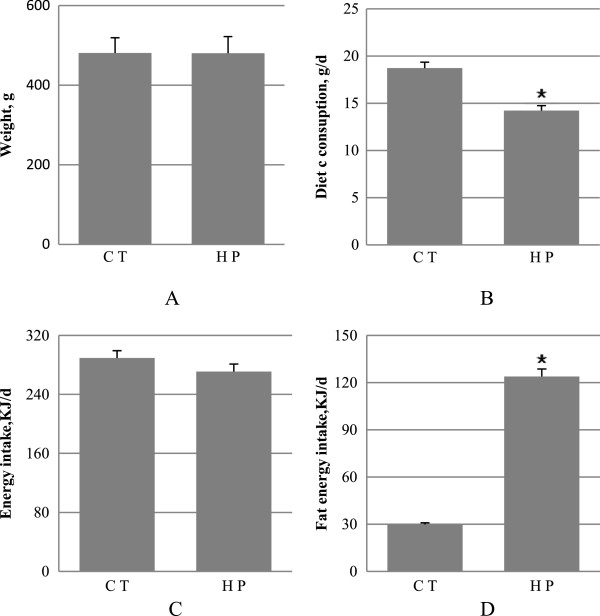
**Weight and feed intake. A**, weight; **B**, feed intake; **C**, energy intake; **D**, fat energy intake. Data are mean ± standard deviation (SD), n = 8 Rats per diet. Significance during the HP compared to CT is indicated by *P < 0.05 (Student’s *t*-test).

Figure 1 summarizes the animal characteristics after 16 weeks of diet. There was no difference in the body weights found between the HP and CT groups. The CT-fed rats took a much larger diet (P < 0.01) than the HP-fed ones. Fat energy intake was higher (P < 0.01) in the HP-fed rats, despite the lack of difference (P = 0.139) in energy intake in the CT group compared with the HP group.

### Serum lipids

**Figure 2:**
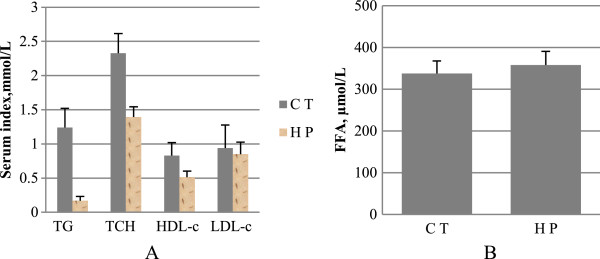
**Indicators of serum lipids. A**, triglycerides (TG), total cholesterol (TCH), high density lipoprotein cholesterol (HDL-c), low density lipoprotein cholesterol (LDL-c); **B**, free fatty acid (FFA). Data are mean ± standard deviation (SD), n = 8 Rats per diet. Significance during the HP compared to CT is indicated by *P < 0.05 (Student’s *t*-test).

As depicted in Figure 2, long-term HP feeding resulted in remarkable decreases in serum TG (P < 0.01), TCH (P < 0.01), and HDL-c (P < 0.01), compared with the CT-fed rats. Serum FFA levels remained unchanged in the HP-fed rats relative to the CT rats (P > 0.05). LDL-c was calculated as previously described [15], for which no difference was shown between the two groups.

### HP-feeding increases hepatic fatty acid oxidation

**Figure 3:**
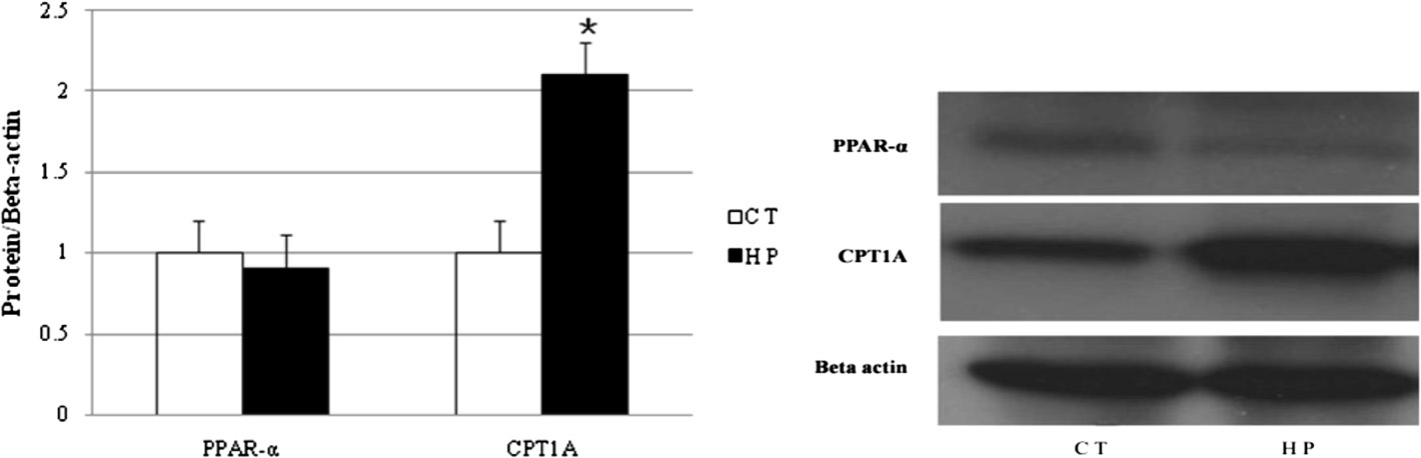
**Western blot of peroxisome proliferator activated receptors-α (PPAR-α, 52 kDa) and carnitine palmitoyltransferase 1A (CPT1A, 88 kDa).** PPAR-α and CPT1A levels were normalised to beta actin (42 kDa). Values are mean from three independent experiments conducted with duplicate treatments, with standard deviations represented by vertical bars. Significance during the HP compared to CT is indicated by *P < 0.05 (Student’s *t*-test).

The expressions of the PPAR-α and CPT1A genes were analyzed in the rats by means of real-time PCR. As depicted in Table 1, a statistically significant up-regulation of PPAR-α and CPT1A mRNA expression was observed in the HP group, compared with the CT group (P < 0.01). The effects of the long-term high-perilla diet on the PPAR-α and CPT1A protein levels were assessed. Consistent with the RNA data, the amount of CPT1A protein increased in the HP group, relative to the CT group. However, the PPAR-α protein did not change (as depicted in Figure 3).

**Table 1:** Relative gene expression levels of hepatic fatty acid oxidation enzymes

**Gene**	**Relative expression**			***P***
	**C T**		**H P**	
PPAR-α	0.321 ± 0.144		0.869 ± 0.435*	<0.001
CPT1A	0.248 ± 0.105		0.46 ± 0.214*	<0.001

Data are mean ± standard deviation (SD), n = 8 Rats per diet. Significance during the HP compared to CT is indicated by *P < 0.05 (Student’s *t*-test).

### HP-feeding induces changes in hepatic lipid synthesis gene expression

**Figure 4:**
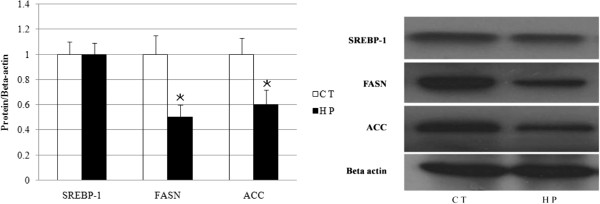
**Western blot of Sterol Regulatory element binding protein-1 (SREBP-1, 65 kDa), Fatty acid synthase (FASN, 273 kDa) and acetyl coenzyme A carboxylase (ACC, 265 kDa) were normalised to beta actin (42 kDa).** Values are mean from three independent experiments conducted with duplicate treatments, with standard deviations represented by vertical bars. Significance during the HP compared to CT is indicated by *P < 0.05 (Student’s *t*-test).

The real-time PCR analysis shows that the mRNA content of SREBP-1, a key transcription factor involved in the regulation of lipogenesis, was higher in the livers of the HP-fed rats than the CT rats (Table 2). However, there were no differences in the SREBP-1 protein levels between the two groups. The mRNA levels of FASN and ACC, two major enzymes involved in fatty acid synthesis, significantly increased in the HP-fed rats, relative to the CT rats. However, the protein levels of FASN and ACC exhibited reverse results. As depicted in Figure 4, the amounts of FASN and ACC protein decreased in the HP group compared with the CT group.

**Table 2:** Relative gene expression levels of hepatic fatty acid synthesis

**Gene**	**Relative expression**			***P***
	**C T**		**H P**	
ACC	0.455 ± 0.153		0.989 ± 0.375*	<0.001
FASN	0.145 ± 0.087		0.404 ± 0.191*	<0.01
SREBP-1	0.372 ± 0.185		0.774 ± 0.327*	<0.01

Data are mean ± standard deviation (SD), n = 8 Rats per diet. Significance during the HP compared to CT is indicated by *P < 0.05 (Student’s *t*-test).

### Rats fed with high-dose perilla diet display insulin resistance

**Figure 5:**
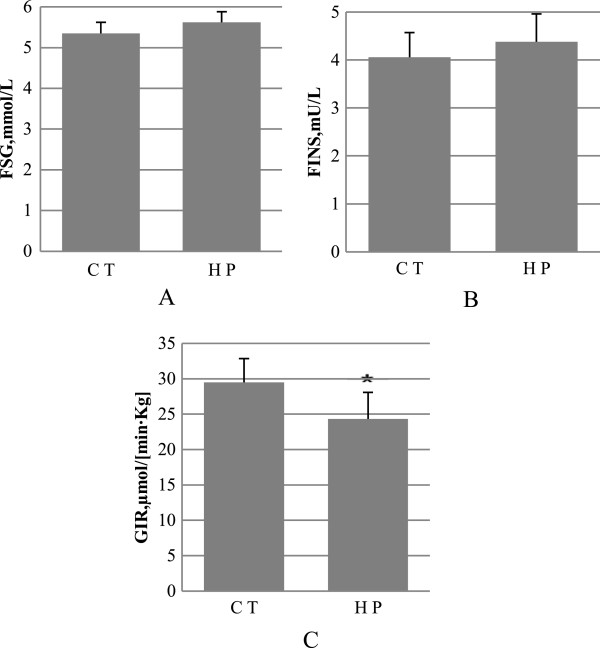
**Insulin sensitivity. A**, fasting serum glucose (FSG); **B**, fasting insulin (FINS); **C**, glucose infusion rat. Data are mean ± standard deviation (SD), in **(A)** and **(B)** n = 8 rats per diet, in **(C)** n = 4 rats per group. Significance during the HP compared to CT is indicated by *P < 0.05 (Student’s *t*-test).

As shown in Figure 5, the insulin and glucose levels in the fasted rats were similar between the CT and HP groups. After application of the euglycemic-hyperinsulinemic clamp, it was found that the HP-fed rats exhibited insulin resistance after fed for 16 weeks, as shown by the higher glucose infusion rate upon extron insulin stimulation in comparison with the CT rats.

### Intrahepatic lipids

**Figure 6:**
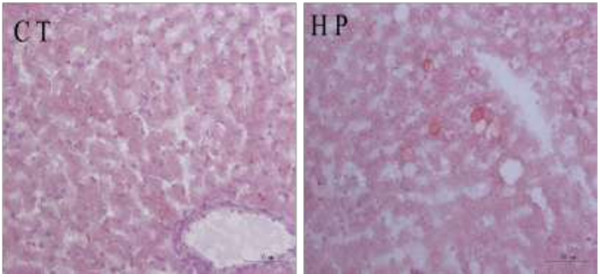
Oil red O staining of liver (original magnification, 200×).

Histological analysis revealed that there were almost no lipid droplets in the livers of the HP-fed rats compared with the CT rats (Figure 6). These observations were confirmed by the biochemical analysis, which showed that hepatic fatty oxidation was up-regulated both in terms of the mRNA and protein levels, and hepatic lipid synthesis was down-regulated in the protein levels after HP feeding.

## Discussion

Being one of the richest sources of ALA, perilla oil consists of 69.11% ALA. After a 16-week dietary treatment with a diet rich in perilla oil, the level of lipids in the serum lowered significantly and hepatic fatty oxidation improved. In contrast, insulin sensitivity, as measured by a euglycemic-hyperinsulinemic clamp, was decreased by the perilla oil-rich diet.

Excessive ALA intake did not cause significant increases in body weight or total body fat in the HP group [16], which is similar to the role of EPA/DHA used to prevent obesity and overweightness [17]. Contrarily, after high-fat diet derived from lard or soybean oil were fed to rats or mice, the results showed that the animals had severe intra-abdominal fat and fat deposition, and their body weights were much higher than those of the control group [18-20]. These results suggest that perilla may affect triglyceride deposition in adipose tissue and fat cell activities (such as cell differentiation and cytokine secretion).

In the present study, the serum levels of triglycerides and total cholesterol of rats in the HP group decreased by 86.5 and 40.2%, respectively, compared with those in the control group. In accord with our data, when the mice were respectively fed a high-ALA diet (12% ALA and 4% LA) and a high-LA diet (12% LA and 3% ALA), the serum levels of total cholesterol of the mice fed with the high-dose ALA decreased significantly after 35 days of feeding [21]. Clinical trials demonstrated that patients with metabolic syndrome had a daily ALA intake of 3.5 g/d on their body weight, and their serum levels of triglycerides and total cholesterol decreased significantly after six months of dietary intervention [22]. Therefore, the data suggest taking high doses of ALA was effective in their accepted role of lowering TG and TCH, and this method may be used to treat patients to whom statins are clinically not applicable. However, the n-3 PUFAs currently used mainly originate from marine products (EPA/DHA) [23-25], while few studies have focused on ALA. In addition, the serum levels of high-density lipoprotein cholesterol in the HP group were significantly lower than those in the control group. The reason for this result may be that peripheral cholesterol does not require being transported to the liver, due to cholesterol reduction.

Liver plays a central role in maintaining energy balance and contributing to energy storage in the fed state. The results of the present study show that, compared with the control group, the hepatic mRNA levels of PPAR-α and CPT1A in the HP group increased by 171 and 85%, respectively, with a significant increase in hepatic CPT1A protein expression. However, no difference in hepatic PPAR-α protein expression was shown, suggesting that the high-perilla intake enhanced β-oxidation. 3 T3-L1 cells were treated with ALA, and by DNA microarray analysis the results show that a 1.7-fold increase occurred in the CPT-1a gene expression [26]. In animal experiments, it has been proven that ALA may enhance fatty acid oxidation. When rats were fed with flaxseed oil and perilla oil, the mRNA expression levels of hepatic CPT-I and CPT-II significantly increased, but no effect was shown in terms of fatty acid synthesis [27]. Gonzalez-Manan D. fed rats with chia (Salvia hispanica) and rosa mosqueta (Rosa rubiginosa) rich in ALA, and found significant increases in the PPAR-α transcript level and CPT1A protein expression level after 21 days of feeding [28]. In the present study, the mRNA expressions of hepatic SREBP-1, FASN and ACC of rats in the HP group were significantly higher, while the protein expressions of FASN and ACC were significantly lower, compared with those of the control group. In addition, the SREBP-1 protein expression showed no significant difference between the two groups. These results indicate that the high-dose ALA inhibited fat synthesis by regulating the translation pathway. SREBP-1 has been found to be a positive transcriptional regulator of the cytosolic lipogenic enzymes, which work in sequence to citrate carrier (CIC). Direct evidence was provided that PUFA decrease CIC gene promoter activity, the CIC transcriptional suppression by PUFA was clearly mediated by the SRE/SREBP-1 regulatory system as its effects on CIC promoter-driven luciferase activity was abolished by mutations in the SRE site of the CIC gene [29-31]. However, the rats in the HP group were given a long-term diet with high energy, and excessive fat deposition did not occur in the liver, due to an increase in fatty acid oxidation and a decrease protein expression of key enzymes in fat synthesis. This finding is in agreement with the observation, obese Zucker rats were fed a diet containing 4% ALA, and hepatic fat accumulation was also significantly inhibited after one month of feeding [32].

In the present study, the mass proportion of ALA in the high-fat diet was 15.74%, and the data from the 16-week clamp experiment show a significant reduction in insulin sensitivity in the HP group. In our previous studies, rats were fed a high-fat diet with the same proportion of perilla, and their insulin sensitivity indexes also decreased significantly after eight weeks of feeding [33]. There have been very few studies in this area to directly compare our data to. However, in terms of application in humans, one study found that an intake of high-dose long-chain n-3 PUFAs (5–8 g/d) increased the blood glucose level in patients with type II diabetes [34]. The reason for this insulin resistance caused by long-term excessive ALA may be that the activity of fatty acid β-oxidation leads to acetyl-CoA accumulation, then pyruvate dehydrogenase activity inhibited by excessive intracellular acetyl-CoA causes citric acid accumulation. As a potential inhibitor of phosphofructokinase, citric acid blocks glucose oxidation in the initial stage, resulting in a decrease in the glucose transportation rate of GLUT4 [35]. Many studies have shown that an excessive saturated fat intake caused an increase of FFA, and elevated FFA had a “toxic” effect on the body, causing damage to the pancreatic beta cell function, promoting cell apoptosis, and resulting in impaired glucose-stimulated insulin secretion [36]. Male Sprague–Dawley rats were fed a chow-diet or a diet high in saturated fat or PUFA (derived from lard and coconut oil or pufa derived from safflower oil). The results show that after 8 weeks, both high-fat diets increased the plasma FFA by 30% [37]. However, excessive ALA intake did not cause elevated serum FFA levels of the rats in the HP group.

After long-term excessive perilla intake, no significant changes were shown in the body weight, hepatic fat deposition or intra-abdominal fat of the rats. In addition, a significant reduction was seen in the serum lipid level, as well as significant increases in the expressions of key enzymes in fatty acid oxidation, along with significant decreases in the protein expressions of key enzymes in fatty acid synthesis. However, the experimental results of the insulin sensitivity reduction will incite interest in researchers studying ALA dosage. More studies regarding the functions and mechanisms of ALA in the bodies of animals and their verification in the human body must be conducted in order to determine the most effective doses of ALA for prevention and treatment.

## Conclusions

We have observed that excessive perilla oil intake may significantly lower serum lipids, strengthen hepatic fatty acid oxidation, and inhibit hepatic fatty acid synthesis, but lead to insulin resistance in rat. Further studies are needed to elucidate the precise mechanism of ALA in the bodies of animal and their verification in the human body must be conducted in order to determine the most effective dose of ALA for prevention and treatment.

## Materials and methods

### Animals and diet

This study was conducted in conformity with the policies and procedures of the Institutional Animal Care and Use Committee of Laboratory Animal Center. Six-week-old male Sprague–Dawley (SD) rats were obtained from the Laboratory Animal Centre of the Academy of Military Medical Sciences(Dong da jie 20^th^, Beijing, China). The rats were housed in groups of four per cage at 22°C with a 12 h light/dark cycle (light period: 6:00–18:00 h) and given free access to diet and water. After acclimatization at the facility for one week, the rats were given ad libitum access to one of two diets for 16 weeks (n = 8 for each group), i.e. the control (CT) diet or a diet high in perilla oil (HP). As listed in Table 3, the control diet consisted of 20.2% calories from protein, 10.31% calories from fat, and 69.49% calories from carbohydrates. The HP diet consisted of 19.67% calories from protein, 45.73% calories from perilla, and 34.6% calories from carbohydrates. The compositions of the diets are listed in Table 4. The fatty acid compositions of the diets are listed in Table 5.

**Table 3:** Dietary energy levels and energy percentages

**Group**	**KJ/g**	**Fat: Protein: Carbohydrate**
C T	15.70	10.31%: 20.20%: 69.49%
H P	19.35	45.73%: 19.67%: 34.59%

**Table 4:** Feed formulas

**C T**		**H P**	
Ingredients	g/100 g	Ingredients	g/100 g
Corn starch	52	Corn starch	20
Maltodextrin	3.2	Maltodextrin	10
Sugar	10	Sugar	10
Soybean Oil	4.3	Perilla Oil	23.5
Casein	19.0	Casein	22.7
Powdered Cellulose	4.7	Powdered Cellulose	5.7
MgO	0.085	MgO	0.102
NacL	0.35	NacL	0.42
Potassium Citrate	0.9	Potassium Citrate	1.08
KSO_4_	0.5	KSO_4_	0.6
Dicalcium Phosphate	2.4	Dicalcium Phosphate	2.88
Mineral Mix	1.625	Mineral Mix	1.95
Vitamin Mix	0.05	Vitamin Mix	0.06
Calcium Carbonate	0.3	Calcium Carbonate	0.36
DL-Methionine	0.35	DL-Methionine	0.42
Choline Bitartrate	0.2	Choline Bitartrate	0.24
Yellow Dye	0.001	Red Dye	0.001

**Table 5:** Types and levels of dietary fatty acids

**Fatty acids**	**Fatty acids in diet, g/100 g**	
	**C T**	**H P**
C16:0	0.49	1.56
C18:0	0.18	0.39
C18:1	0.98	2.91
C18:2	2.37	2.90
C18:3	0.28	15.74
Total	4.30	23.50

### Serum parameters

Upon completion of the experiments, all rats were weighed and blood was collected from the anesthetized animals into blood collection tubes after an overnight fast (12 h). After standing for 30 min, the serum was prepared by centrifugation of blood at 1000 × g for 10 min at 4°C and stored at −80°C until analysis. Serum triglyceride (TG), total cholesterol (TCH), high density lipoprotein-cholesterol (HDL-c) and glucose (GLU) were measured by means of enzymatic and colorimetric methods, using assay kits (Sinopharm Chemical Reagent Beijing Co., Ltd, China). Free fatty acid (FFA) and insulin (INS) concentration were determined using rat FFA and insulin ELISA kits (Novateinbio INC), respectively. LDL cholesterol was calculated according to the method of Friedewald et al. [15].

### Real-time quantitative PCR

cDNA was prepared by reverse transcription of six micrograms of total RNA using the Kit Reverse transcription System. Real-time qPCRs were performed with an IQ5 instrument and software. RNA levels were determined by analyzing the changes in SYBR Green Ifluorescence during PCR, according to the manufacturer's instructions. β-actin was amplified in parallel, and the results were used for normalization. The correct size of the PCR product was confirmed by electrophoresis on a 2.5% agarose gel stained with ethidium bromide. The purity of the amplified PCR products was determined by melting point analysis. The primers and gene details are summarized in Table 6.

**Table 6:** Primers for analysis of transcription level of gene expression

**Gene**	**Accession no.**	**Primer**	**Tm, °C**	**Amplicon size,bp**
PPAR-α	NM_013196.1	F 5'-GGTCATACTCGCAGGAAAG-3'	53	151
		R 5'- GCAGCAGTGGAAGAATCG-3'		
CPT1A	NM_031559.2	F 5'-GCCAGACGAAGAACATTG-3'	53	171
		R 5'-CCTTGACCATAGCCATCC-3'		
ACC	NM_022193	F 5'-TTGGTGCTTATATTGTGGATGG-3'	50	127
		R 5'-ATGTGCCGAGGATTGATGG-3'		
FASN	NM_017332.1	F 5'-GGTAGGCTTGGTGAACTGTCTC-3'	55	201
		R 5'-TCTAACTGGAAGTGACGGAAGG-3'		
SREBP-1	XM_213329	F 5'-CATCAACAACCAAGACAGTG-3'	50	126
		R 5'-GAAGCAGGAGAAGAGAAGC-3'		
ACTIN	NM_031144.2	F 5'–CCC ATC TAT GAG GGT TAC GC-3'	50	150
		R 5'-TTT AAT GTC ACG CAC GAT TTC-3'		

### Immunoblotting

Total proteins were prepared from livers (~100 mg), as previously described [38]. The proteins were resolved by SDS-PAGE at 80 V for 30 min, followed by 120 V for 100 min, then transferred to polyvinylidene difluoride membranes at 250 mA for 2 h. Membranes were blocked for 1 h at room temperature, with PBS containing 0.1% tween and 5% skim milk powder, and incubated with mouse monoclonal anti-PPARα antibodies (1:5000, 52 kDa), mouse monoclonal anti-CPT1A antibodies (1:5000, 88 kDa), mouse monoclonal anti-SREBP-1 antibodies (1:5000, 65 kDa), rabbit monoclonal anti-fatty acid synthase antibodies (1:5000, 273 kDa), rabbit monoclonal anti-acetyl coenzyme A carboxylase antibodies (1:5000, 265 kDa), and mouse monoclonal anti-beta actin antibodies (1:5000, 42 kDa) (all antibodies were purchased from Abcam Inc., USA), overnight at 4°C, then washed for 3 × 10 min with PBS containing 0.1% tween. After incubation with the appropriate secondary antibody for 1 h at room temperature, the membranes were washed for 3 × 10 min with PBS. Then the bands were visualized by ECL and quantified densitometry.

### Euglycemic-hyperinsulinemic clamp study

After 16 weeks of feeding, the clamp was done. The rats were anesthetized with isoflurane (Sinopharm Chemical Reagent Beijing Co., Ltd, China) after fasting overnight. The left common carotid artery and right jugular vein were catheterized as previously described [39]. The clamps were then performed 4–5 d later after complete recovery of the rats from the operation. Rats (n =4 per group) fasted for 12 h, were infused with human insulin (Humulin R, Novo Nordisk) into the venous circulation, at a rate of 4 mU · kg-1 min-1 for 2 h. Throughout the infusion, the carotid artery was assessed every 10 min using a blood glucose meter(One Touch Uitra, Lifescan). Titration of glucose continues until stable glucose readings are achieved. Stable glucose levels over a time course of a minimum of 30 min. Glucose levels and glucose infusion rates during this stable period are recorded and reported. Results provide an index of whole body insulin sensitivity.

### Oil red O staining

For the detection of neutral lipids, portions of liver were rapidly frozen in liquid nitrogen and embedded in Tissue-Tek. 5 μm cryosections were mounted on the microscope slides and air-dried for 2 h. After fixation in 4% neutral formaldehyde for 10 min, sections were stained with oil red O, with 0.5% oil red O dissolved in propylene glycol for 10 min at 60°C. The sliced sections were then counterstained.

### Statistical analysis

The results are presented as means ± standard error. The differences among two diet groups were analyzed by Student’s *t*-test within the SAS version 9.0 statistical package. Differences were considered to be significant at p < 0.05.

## Abbreviations

TCH: Total cholesterol; TG: Triglyceride; HDL-c: High density lipoprotein cholesterol; LDL-c: Low density lipoprotein cholesterol; FFA: Free fatty acid; ALA: Alpha-linolenic acid; PPAR-α: Peroxisome proliferator activated receptors-α; CPT1A: Carnitine palmitoyltransferase 1A; FASN: Fatty acid synthase; SREBP-1: Sterol Regulatory element binding protein-1; ACC: acetyl coenzyme A carboxylase.

## Competing interest

The authors declare that they have no competing of interest.

## Authors’ contributions

TZ and SZ performed most of the experiments and prepared the manuscript and should be regarded as co-first authors. WL, LM carried out the animal studies and biochemical analysis. MD carried out data collection and analysis. RL helped to draft the manuscript. YL conceived the study, participated in its design and coordination, corrected the manuscript and supervised the project. All authors read and approved the final manuscript.
